# Omicron: Master of immune evasion maintains robust ACE2 binding

**DOI:** 10.1038/s41392-022-00965-5

**Published:** 2022-04-07

**Authors:** Markus Hoffmann, Lu Zhang, Stefan Pöhlmann

**Affiliations:** 1 Infection Biology Unit, German Primate Center—Leibniz Institute for Primate Research, Göttingen, Germany; 2 Georg-August-University Göttingen, Göttingen, Germany

**Keywords:** Microbiology, Infectious diseases

The spike (S) protein of the SARS-CoV-2 Omicron variant is highly mutated but the impact of these mutations on viral entry into cells and its inhibition by antibodies has been unclear. A recent study published in Science by Mannar et al. now shows that these mutations are compatible with robust ACE2 binding and allow for efficient evasion of neutralizing antibodies.

**Fig. 1:**
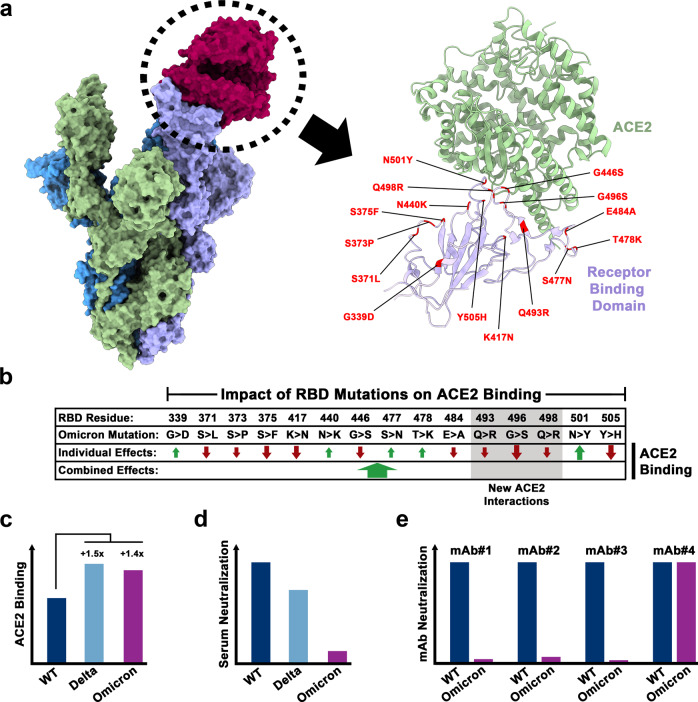
ACE2 interactions and resistance against antibody-mediated neutralization of the Omicron spike protein. **a** Left panel: Model of the trimeric SARS-CoV-2 spike protein bound to ACE2 (red). Right panel: Close-up on the binding interface of the receptor binding domain (RBD) of the SARS-CoV-2 spike protein (violet) and ACE2 (green). RBD residues that are mutated in the Omicron variant sublineage (BA.1) are highlighted (red). **b** Individual and combined impact of Omicron-specific RBD mutations. Omicron residues that establish new interactions with ACE2 are highlighted (gray). **c** Relative ACE2 binding by the spike proteins of wild-type (WT) SARS-CoV-2 and SARS-CoV-2 variants Delta and Omicron. **d** Convalescent and vaccinee sera exhibit low neutralization potency against the Omicron variant. **e** The Omicron variant is resistant against most monoclonal antibodies (mAbs) that neutralize WT SARS-CoV-2 with high potency.

The emergence of SARS-CoV-2 variants has become a hallmark of the COVID-19 pandemic. Variants of concern (VOC) harbor mutations in the viral S protein that can increase transmissibility, potentially by promoting S protein binding to the cellular receptor ACE2. Alternatively, mutations may alter epitopes of neutralizing antibodies, enabling the virus to infect convalescent or vaccinated individuals. The Delta variant dominated globally until the winter season 2021 when it was abruptly outcompeted by the emerging Omicron variant, which experienced an explosive global spread. Several features of the Omicron variant are incompletely understood, including its origin and the reason why the Omicron variant is less well able than the Delta variant to infect the lung and to cause severe disease. The Omicron S protein harbors at least 3–4 times more mutations than the S proteins of other VOCs and several of these mutations are known to reduce ACE2 binding. Mannar and coworkers demonstrate that these mutations are compensated by others, which establish new ACE2 contacts (Fig. 1a, b), resulting in overall robust ACE2 binding of the Omicron S protein (Fig. 1c).^1^ Further, they show that mutations in the Omicron S protein confer efficient evasion of antibody-mediated neutralization (Fig. 1d, e).^1^ In the following we will summarize their key findings.

Surface plasmon resonance analyses showed that Omicron S protein bound to ACE2 with increased affinity as compared to the S protein of a virus circulating earlier in the pandemic, Wuhan Hu-1 (subsequently termed WT), and with similar affinity as the S protein of the Delta variant (Fig. 1c).^1^ The Omicron receptor binding domain (RBD) contains mutation K417N, which decreases ACE2 binding by abrogating a salt bridge to ACE2 residue D30. Further, mutagenic analyses by other groups had shown that the majority of the other mutations present in the Omicron RBD also reduced receptor binding (Fig. 1a, b), raising the question why Omicron S still bound to ACE2 with high affinity. Employing cryo-electron microscopic analysis of the Omicron S protein trimer in complex with ACE2, Mannar et al. found that compensatory mutations were responsible.^1^ Thus, some of the amino acid substitutions in the Omicron RBD allowed for the formation of new bonds with ACE2, including a salt bridge between RBD residue R498 and ACE2 residue D38 and a hydrogen bond between RBD residue S496 and ACE2 residue K353, resulting in an overall increase of ACE2 binding (Fig. 1a, b).^1^

The impact of mutations in the Omicron S protein on evasion from neutralizing antibodies was studied using pseudotyped viruses. Most monoclonal antibodies that inhibited the WT S protein failed to efficiently block Omicron (sublineage BA.1) S protein with the exception of antibody S309 (Sotrovimab),^1^ which recognizes an epitope that is highly conserved among sarbecoviruses (Fig. 1e). Further, the Omicron S protein was less well neutralized by antibodies induced upon vaccination and infection as compared to the S proteins of WT and Delta variant, respectively (Fig. 1d).^1^ These findings indicate that the mutations in the Omicron S protein alter many epitopes recognized by neutralizing antibodies, underlining the high plasticity of the S protein/ACE2 interface.

The robust binding of Omicron S protein to ACE2 due to compensatory mutations, which was also reported by others,^2,3^ translates into efficient usage of human ACE2 and ACE2 orthologues from different animal species, including rodents, for host cell entry.^4^ It has even been suggested that the Omicron variant might have originated from rodents, a scenario that is also supported by the nature of the base substitutions in the Omicron genome and the presence of mutations in the Omicron S protein that promote binding to murine ACE2. However, other possibilities are also being discussed, including emergence of the Omicron variant in persistently infected, immunocompromised human patients.

The robust ACE2 binding of the Omicron S protein suggests that receptor interactions might not limit viral cell tropism. However, the Omicron S protein requires higher ACE2 levels for membrane fusion than the WT virus, likely due to slower transition into the so called 2- or 3-RBD-up conformations, and inefficient lung infection by the Omicron variant might be due to low ACE2 expression.^2^ Alternatively, the choice of S protein-activating host cell protease might be responsible. Thus, the Omicron S protein, unlike the S proteins of WT virus and all previous VOCs, fails to use the serine protease TMPRSS2 for efficient S protein activation.^5^ Although the Omicron variant employs the cysteine protease cathepsin L for infection of nasal epithelium, an unknown, possibly TMPRSS2-related serine protease promotes infection of airway epithelium but might be absent from alveolar type 2 cells, which could account for the inability of the Omicron variant to infect these cells and to efficiently spread in lung tissue.^5^

Mannar et al. suggest that the deletion Δ143-145 in the amino terminal domain (NTD) of the S protein rendered the Omicron S protein resistant against the NTD-specific antibodies tested. Other studies reached similar conclusions, also suggesting an important role of G142D in antibody evasion and demonstrating that the mutations in the NTD had reconfigured this domain, substantially changing its antigenic properties.^2^^,3^ The impact of the Omicron-specific mutations on the overall RBD structure were found to be less prominent although many surfaces were altered, resulting in altered antibody binding sites and immune evasion. Importantly, evasion from neutralizing antibodies seems to be largely accountable for the explosive spread of the Omicron variant, since Omicron patients apparently do not exhibit higher viral loads and a transmission advantage of the Omicron variant is mainly seen for vaccinated as compared to unvaccinated contacts.

Collectively, the results by Mannar et al. reveal how the Omicron variant can evade antibody responses with unprecedented efficiency without compromising ACE2 binding although it remains to be determined which mutations preclude efficient spread in the lung and how.
